# Weight Stigma Model on Quality of Life Among Children in Hong Kong: A Cross-Sectional Modeling Study

**DOI:** 10.3389/fpsyg.2021.629786

**Published:** 2021-04-22

**Authors:** Chia-Wei Fan, Chieh-hsiu Liu, Hsin-Hsiung Huang, Chung-Ying Lin, Amir H. Pakpour

**Affiliations:** ^1^Department of Occupational Therapy, AdventHealth University, Orlando, FL, United States; ^2^National Cheng Kung University Hospital, College of Medicine, National Cheng Kung University, Tainan, Taiwan; ^3^Department of Statistics and Data Science, University of Central Florida, Orlando, FL, United States; ^4^Institute of Allied Health Sciences, College of Medicine, National Cheng Kung University, Tainan, Taiwan; ^5^Department of Occupational Therapy, College of Medicine, National Cheng Kung University, Tainan, Taiwan; ^6^Department of Public Health, National Cheng Kung University Hospital, College of Medicien, National Cheng Kung University, Tainan, Taiwan; ^7^Department of Rehabilitation Sciences, Faculty of Health and Social Sciences, The Hong Kong Polytechnic University, Hung Hom, Hong Kong; ^8^Social Determinants of Health Research Center, Research Institute for Prevention of Non-communicable Diseases, Qazvin University of Medical Sciences, Qazvin, Iran; ^9^Department of Nursing, School of Health and Welfare, Jönköping University, Jönköping, Sweden

**Keywords:** Asia, children, quality of life, structural equating modeling, weight-related stigma

## Abstract

We proposed a model to examine the relationship among different types of weight-related stigmas and their relationship to quality of life (QoL). We recruited 430 dyads of elementary school children [mean age = 10.07 years; n_boy_ = 241 (56.0%); n_overweight_ = 138 (32.1%)] and their parents. Parents completed QoL instruments about their children assessing generic QoL and weight-related QoL. Children completed QoL instruments assessing generic QoL and weight-related QoL and stigma scales assessing experienced weight stigma, weight-related self-stigma, and perceived weight stigma. Experienced weight stigma was significantly associated with perceived weight stigma, and in turn, perceived weight stigma was significantly associated with weight-related self-stigma. However, experienced weight stigma was not directly associated with weight-related self-stigma. In addition, experienced stigma was negatively associated with both child-rated and parent-rated QoL. Perceived weight stigma was associated only with parent-rated weight-related QoL but not child-rated QoL. Self-stigma was associated with child-rated QoL but not parent-rated QoL. Moreover, perceived weight stigma and weight-related self-stigma were significant mediators in the association between body weight and children's QoL; experienced weight stigma was not a significant mediator. The study findings can be used to inform healthcare providers about the relationship among different types of stigmas and their influence on child-rated and parent-rated QoL and help them develop interventions to address the global trend of overweight/obesity in youth and pediatric populations.

## Introduction

The trend in obesity in youth and pediatric populations significantly increased from 1999 to 2000 through 2015–2016 (Hales et al., 2017). It has reached epidemic levels in the United States (Sanyaolu et al., 2019). Apart from the United States, the rising global prevalence of obesity was also found in Africa and Asia (Güngör, 2014). Particularly among the Asian population, the risk may begin to increase at a lower body mass index (BMI) compared to other races and Hispanic-origin populations (Jafar et al., 2005; Zheng et al., 2011). Children with obesity are likely to stay with obesity into adulthood and have a greater risk of suffering from weight-related health problems (Sahoo et al., 2015). Many studies have shown the association between childhood obesity and physical (e.g., Type 2 diabetes, high blood pressure, heart disease, etc.) and psychosocial (e.g., depression, anxiety, socially isolating, etc.) problems (Bass and Eneli, 2015; Bacha and Gidding, 2016).

Overweight/obesity has been linked to impaired quality of life (QoL) or health-related QoL (HRQoL). QoL can be evaluated with generic and disease-specific assessment tools. The prior assessments contain general QoL items that apply to a wider variety of populations and can be used for comparison across various conditions. The later assessments contain items with specific characteristics particularly relevant to a disease or a health condition, which can be more responsive to minimal clinical changes (Zeller and Modi, 2009). Because generic and condition-specific (e.g., weight concerns) assessments evaluate different QoL perspectives, previous studies have recommended adopting both types of assessments to achieve a comprehensive understanding of QoL (Kolotkin et al., 2006; Tsiros et al., 2009; Vos et al., 2012). Moreover, prior studies have found that childhood overweight was negatively associated with a broad range of health indicators, including QoL (Swallen et al., 2005; Pinhas-Hamiel et al., 2006; Riazi et al., 2010; Wille et al., 2010; Al-Akour et al., 2011; Hamzaid et al., 2011; Kuhl et al., 2012; Pratt et al., 2012; Halfon et al., 2013; Jansen et al., 2013; Buttitta et al., 2014; Morrison et al., 2015; Rankin et al., 2016; Meixner et al., 2020). Therefore, understanding QoL is a key element essential for childhood obesity.

Unfortunately, impaired QoL is not solely due to overweight/obesity. Other psychosocial factors may cause QoL impairment for children. Specifically, weight stigma, which has been highly prevalent among child populations over decades (Puhl et al., 2008; McCormack et al., 2011; Puhl and Lessard, 2020; Fields et al., 2021), also contributes to lowered QoL. A recent study indicated that almost a quarter to a half of children had been bullied due to their body weight (Thompson et al., 2020). Similarly, Pakpour et al. (2019b) found that weight-related self-stigma was significantly associated with perceived weight stigma and QoL among 287 third to sixth graders, regardless of their weight status. Their results additionally showed that weight-related self-stigma was significantly associated with both generic QoL (assessed using Kid-KINDL) and weight-related QoL [assessed using Sizing Me Up (SMU)]. Moreover, its association with weight-related QoL was stronger than that with generic QoL. Therefore, healthcare providers importantly need to have in-depth knowledge of the mechanism between weight stigma and QoL.

Weight stigma is defined as “negative weight-related attitudes and beliefs that are manifested by stereotypes, bias, rejection, and prejudice” (Puhl and Latner, 2007, p. 558). It can be categorized into three different types on the personal level: experienced stigma, perceived stigma, and self-stigma (Alimoradi et al., 2020). Experienced stigma results when an individual receives actual discrimination toward himself or herself. Perceived stigma occurs when an individual believes how most people view the stigmatized group in general. Self-stigma occurs when an individual internalizes the stigma belief and accepts the discrimination toward his/her personal characteristic. Gmeiner and Warschburger (2020) confirmed the association between experienced weight stigma and weight-related self-stigma using a longitudinal study design on children. They found that experiencing more weight-related teasing is a risk factor for children to develop weight-related self-stigma.

The associations between weight stigma and QoL have been documented for the three types of weight stigma. Previous studies found that adolescents who experienced weight stigma had low levels of psychological QoL (Greenleaf et al., 2014). Similarly, children who experienced weight stigma reported poor psychological QoL (Gunnarsdottir et al., 2012) and overall HRQoL subsequently (Jensen et al., 2014). A meta-analysis shows that perceived weight stigma and weight-related self-stigma were negatively associated with psychological well-being (Alimoradi et al., 2020). The association between weight-related self-stigma and QoL was also verified by Pakpour et al. (2019b). Another previous study examining the relationship between stigma and psychological well-being found that the experienced stigma and self-stigma are more important than weight status in explaining psychological functioning in childhood (Zuba and Warschburger, 2017).

Moreover, a recent study proposed a mediation model to consider the relationship among weight status, experienced stigma, and HRQoL on 600 community children aged 8–11 years (Guardabassi et al., 2018). The results indicated that the increased weight-related experienced stigma, rather than weight status, negatively affected both global and domain-specific HRQoL in middle childhood (Guardabassi et al., 2018).

Although the association between weight-related stigma and QoL has been reported, no empirical evidence has shown how the different types of weight-related stigma contribute to the generic and weight-related QoL. Most studies have examined associations on one type of stigma at a time instead of considering a comprehensive profile of different types of weight-related stigma that might be associated with QoL. Given the lack of evidence about the relationship among different types of stigma in child populations and the need to understand the relationship between stigma and QoL, we propose a model that combines both aims to understand the mechanism behind weight stigma and QoL in child populations. In the current study, we first examine the relationships among all types of stigma (i.e., experienced stigma, perceived stigma, and self-stigma). Then, we examine how the different types of stigma link to parent-rated and children-rated generic and weight-related QoL. Accordingly, we hypothesized that (1) children's experienced stigma may be positively associated with their perceived stigma, and then the perceived stigma may be further positively associated with an internalized belief that links to children's self-stigma [aligning with Gmeiner and Warschburger (2020)]; (2) different types of weight stigma are negatively associated with both parent-rated and child-rated generic and weight-related QoL but in different levels [based on Pakpour et al. (2019b)]; (3) different types of weight stigma are mediators in the association between body weight and QoL (including both parent-rated and child-rated generic and weight-related QoL) in that body weight is positively associated with weight stigma, and subsequently, weight stigma is negatively associated with QoL [congruent with Guardabassi et al. (2018)].

## Methods

### Participants and Procedures

The Human Subject Ethics Review Board in the Hong Kong Polytechnic University (Ref. No.: HSEARS20160824003) approved the study proposal before data collection commenced. Eligible participants (including both the children and one of their parents) who were interested in this study signed a written consent form before participating. Specifically, the authors contacted all the primary schools and some non-government organizations (NGOs) in Hong Kong to seek their willingness to collaborate in the study. Two primary schools and two NGOs agreed to collaborate. Then, teachers and staff in the schools or NGOs helped disseminate the study information to their students or members. After inviting 437 dyads of children and their parents through convenience sampling, 430 dyads participated in the study (response rate: 98.4%). Participants' parents completed the demographic information and two QoL instruments on their children. The children completed a set of self-reported questionnaires, including three weight stigma scales and two QoL instruments. For dyads of children and parents recruited from primary school, all the children completed the questionnaires in a classroom under their teachers' supervision. All the parents completed the questionnaires at home. For dyads of children and parents recruited from NGOs, children and parents completed the questionnaires in a quiet room in the NGOs under a research assistant's supervision without disturbance. Moreover, the children and their parents were separated when they completed the questionnaire.

Eligible participants were identified using the following inclusion criteria: (1) children between 8 and 12 years of age; (2) children were currently studying in a primary school in Hong Kong; (3) children were able to read and write Chinese; (4) children and their parents both agreed to participate in this study voluntarily. Children with any of the following conditions were excluded from the study: (1) having any neurological diseases (e.g., autism spectrum disorder and attention-deficit/hyperactivity disorder); (2) having impairments in cognition; (3) having any physical disability (e.g., amputation).

### Instruments

#### Weight Bias Internalization Scale

The Weight Bias Internalization Scale (WBIS) is a commonly used instrument that measures the extent to which an individual endorses and accepts weight-based stereotypes (Pakpour et al., 2019b; Lin et al., 2020a). There are 11 items rated based on a 5-point Likert scale, where score 1 represents *strongly disagree* and score 5 represents *strongly agree* (Durso and Latner, 2008). A higher score indicates a higher level of weight-related self-stigma. The 11 items were constructed in a single domain representing weight-related self-stigma, which has been supported by confirmatory factor analysis (Pakpour et al., 2019b; Lin et al., 2020a). Moreover, the Chinese WBIS had satisfactory psychometric properties (Wong et al., 2019). The Cronbach's α of the WBIS in the present study was 0.88.

#### Weight Self-Stigma Questionnaire

The Weight Self-Stigma Questionnaire (WSSQ) is another commonly used instrument that measures weight-related self-stigma. Specifically, the WSSQ contains two domains: one assesses weight-related self-stigma (or self-devaluation named by the developer), and another assesses perceived weight stigma (or fear of enacted stigma named by the developer) (Lillis et al., 2010). There are 12 items rated (the first six items assess weight-related self-stigma; the last six items assess perceived weight stigma) based on a 5-point Likert scale. Score 1 represents *strongly disagree*, and score 5 represents *strongly agree* (Lillis et al., 2010). A higher score indicates a higher level of weight-related self-stigma or perceived weight stigma. The Chinese WSSQ had satisfactory psychometric properties (Lin and Lee, 2017). The Cronbach's α of the WSSQ in the present study was 0.91.

#### Experienced Weight Stigma

The Experienced Weight Stigma (EWS) uses 10 dichotomous items (“yes” scores 1, and “no” scores 0; sample item: people behave as if you are inferior because of your weight status) to construct a single construct of experienced stigma on weight bias received by an individual. A higher score indicates a higher level of experienced weight stigma. The EWS (including the Chinese version) had satisfactory psychometric properties (Cheng et al., 2018; Lin et al., 2020b). The Cronbach's α of the EWS in the present study was 0.80.

#### Child- and Parent-Rated Kid-KINDL

The Kid-KINDL is a generic QoL assessment instrument for children aged 8–12 years. The Kid-KINDL includes a parallel child self-report and parent proxy report. Each Kid-KINDL report consists of 24 items embedded in six domains (physical well-being, emotional well-being, self-esteem, family, friends, and school functioning). Each domain has four items, and all items were rated on a 5-point Likert scale. The Likert scale was then linearly transformed to a 0–100 scale to indicate the level of QoL. A higher Kid-KINDL score indicates a higher level of QoL (Ravens-Sieberer and Bullinger, 2000). The Chinese Kid-KINDL had satisfactory psychometric properties (Chan et al., 2014; Lin et al., 2014, 2017; Lin, 2018; Pakpour et al., 2019a). The Cronbach's α of the child-rated Kid-KIND in the present study was 0.81 and that of parent-rated Kid-KINDL was 0.76.

#### Sizing Me Up and Sizing Them Up

SMU (child-rated report) and Sizing Them Up (STU) (parent-rated report) are weight-related QoL assessment instruments for children aged 5–18. SMU consists of 22 items embedded in five domains: emotional (four items), physical (five items), teasing experience (two items), positive attributes (six items), and social avoidance (five items). STU consists of 22 items embedded in six domains: emotional (seven items), physical (five items), teasing experience (three items), positive attributes (four items), mealtime disturbance (two items), and school (one item). All items were rated on a 4-point Likert scale. The Likert scale was then linearly transformed to a 0–100 scale to indicate the level of QoL. A higher SMU or STU score indicates a higher level of QoL (Modi and Zeller, 2008; Zeller and Modi, 2009). Both Chinese SMU and STU had satisfactory psychometric properties (Strong et al., 2017; Lin et al., 2018). The Cronbach's α of the SMU in the present study was 0.82 and that of STU was 0.81.

#### Demographic Information

The participants' demographics were assessed using a parent-rated background information sheet to obtain the participants' age in years, gender (boy or girl), height in centimeters, weight in kilograms, health status (with or without chronic illness), subjective academic standing (good, moderate, or poor), exercise habit (yes or no), and monthly family income [below 5,000 Hong Kong Dollar (HKD), 5,000–9,999 HKD, 10,000-14,999 HKD, 15,000–19,999 HKD, 20,000−24,999 HKD, 25,000–29,999 HKD, 30,000–34,999 HKD, 35,000–39,999 HKD, 40,000–44,999 HKD, 45,000–50,000 HKD, or above 50,000 HKD]. The BMI was then calculated using the height and weight information provided in the background information sheet.

### Data Analysis

**Figure 1:**
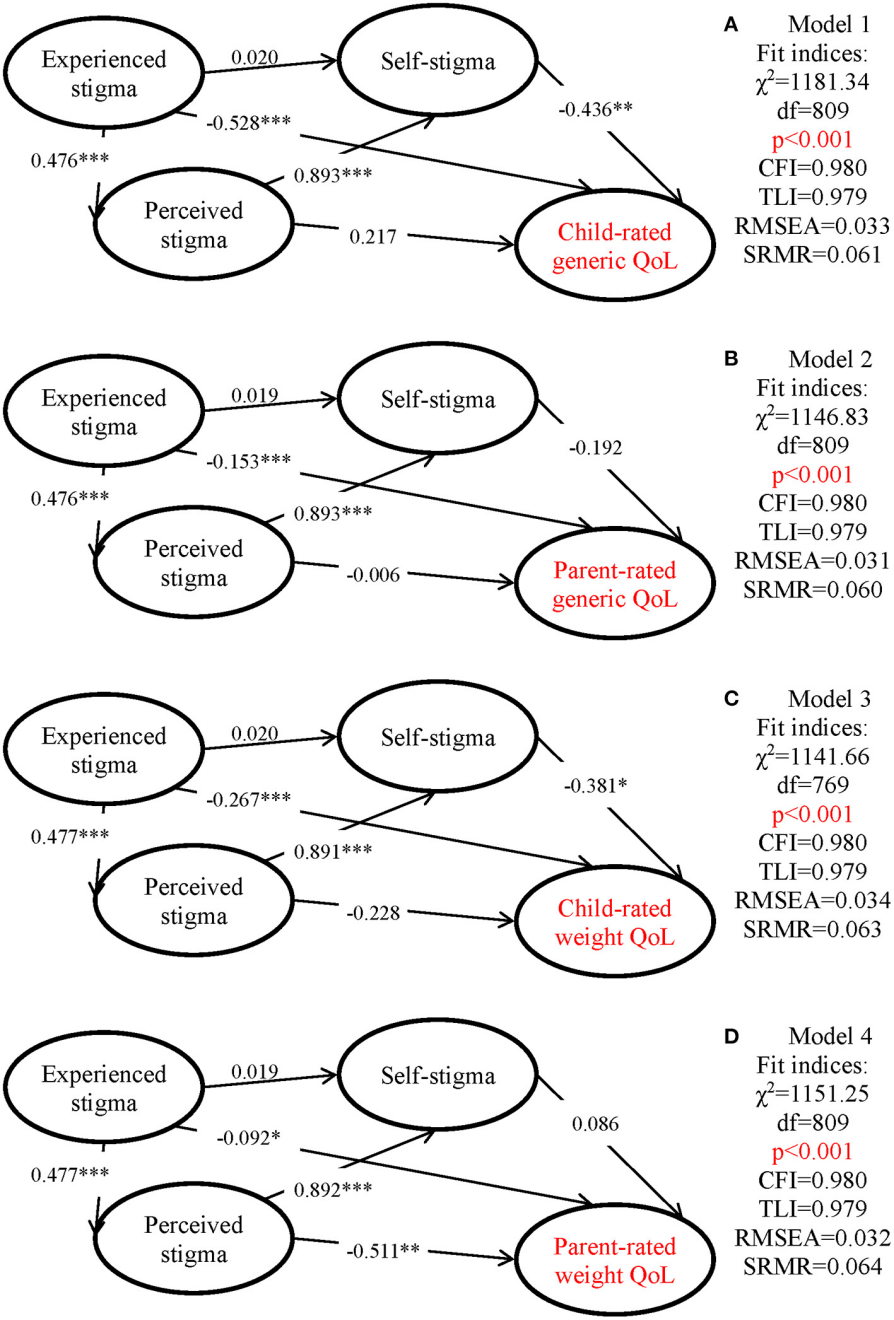
Proposed models evaluating different types of weight bias on quality of life (QoL) with standardized path coefficients. **(A)** Model 1: QoL assessed using child-reported generic instrument (Kid-KINDL). **(B)** Model 2: QoL assessed using parent-reported generic instrument (Kid-KINDL). **(C)** QoL assessed using child-reported weight-related instrument (Sizing Me Up). **(D)** QoL assessed using parent-reported weight-related instrument (Sizing Them Up). All models controlled age, gender, and body mass index. CFI, comparative fit index; TLI, Tucker-Lewis index; RMSEA, root mean square error of approximation; SRMR, standardized root mean square residual. *p < 0.05; **p < 0.01; ***p < 0.001.

Structural equation modeling (SEM) using a diagonally weighted least squares (DWLS) estimator was applied to test our hypothesized theoretical structures. All types of weight stigma (including experienced weight stigma, perceived weight stigma, and weight-related self-stigma) are predictors of QoL; experienced and perceived weight stigma are predictors of weight-related self-stigma; and experienced weight stigma is a predictor of perceived weight stigma (see Figure 1 for the conceptual model). Moreover, the experienced weight stigma was constructed using all EWS items; the perceived weight stigma using items 7–12 in the WSSQ; the weight-related self-stigma using all WBIS items and items 1–6 in the WSSQ; the QoL using different child-rated and parent-rated QoL instruments.

The hypothesized structure was tested in four models. Specifically, all models used the same weight stigma measures but different QoL measures: Model 1 used the child-rated Kid-KINDL; Model 2 used the parent-rated Kid-KINDL; Model 3 used the SMU; and Model 4 used the STU. Additionally, all models have adjusted for age, gender, and BMI. The following fit indices with suggested cutoff were used to determine whether our hypothesized models are supported: comparative fit index (CFI) and Tucker–Lewis index (TLI) >0.9; root mean square error of approximation (RMSEA) and standardized root mean squared residual (SRMR) <0.08. Moreover, a non-significant χ^2^ indicates a good fit between the data and model; however, given that the χ^2^ index is notorious in its oversensitivity to sample size (i.e., χ^2^ easily will be significant in a large sample size such as the size in the present study) (Wu et al., 2015), the fit between the data and model depends on CFI, TLI, RMSEA, and SRMR more.

Referencing the findings of Guardabassi et al. (2018), several mediation models were conducted to explore the mediated effects in different weight stigma types. More specifically, Hayes' Process macro (Model 4) was then carried out to understand the mediated effects (Hayes, 2018) of different types of weight stigma on the association between body weight and QoL. In the Hayes' Process macro, 5,000 bootstrapping samples were generated to examine whether each type of weight stigma is a significant mediator in the association between body weight and QoL. The lower limit of confidence interval (LLCI) and upper limit of confidence interval (ULCI) at 95% were used to examine whether the mediated effect is significant. Specifically, when LLCI and ULCI do not cover 0, the mediated effect is significant. Moreover, age and gender were controlled in all the mediation models.

Moreover, given our sample's relatively wide age range, Pearson correlation coefficients were used to examine the bivariate associations between studied variables, including age, gender, experienced weight stigma, perceived weight stigma, weight-related self-stigma, and all the QoL.

The SEM was conducted by the lavaan package (http://lavaan.ugent.be/) in the R program. Descriptive correlational analyses and Hayes' mediation models were conducted by the IBM SPSS 20.0 (IBM Corp., Armonk, NY).

## Results

The mean (SD) age of the children was 10.07 (1.42), and the genders were generally equally distributed (56% of males). Most children reported having no chronic disease (94.0%) and no exercise habits (93.3%). Moreover, their academic standing was normally distributed (Table 1).

**Table 1:** Participant characteristics.

	**Mean (SD)**	***n* (%)**
**Age (years)**	10.07 (1.42)	
**Gender**		
Male		241 (56.0)
Female		189 (44.0)
**Body mass index (kg/m**^**2**^**)**	18.47 (4.16)	
**Health status**		
Without chronic illness		404 (94.0)
With chronic illness		24 (5.6)
Missing		2 (0.4)
**Subjective academic standing**		
Good		87 (20.2)
Moderate		261 (60.7)
Poor		71 (16.5)
Missing		11 (2.6)
**Exercise habit**		
Yes		23 (5.3)
No		401 (93.3)
Missing		6 (1.4)
**Monthly family income**		
<25,000 HKD		261 (60.7)
>25,000 HKD		148 (34.4)
Missing		21 (4.9)

*HKD, Hong Kong Dollar. 1 USD ≈ 7.8 HKD*.

Table 2 demonstrates the mean and SD of the participants' instrument scores, including weight-related self-stigma, perceived stigma, experienced stigma, and each domain of the QoL instrument. Table 3 further presents the factor loadings of all items embedded in their belonging constructs. In brief, all the loadings are significant and weighted substantially for each construct. Moreover, Table 4 presents the correlation coefficients in every two studied variables (i.e., age, gender, BMI, three types of weight stigma, and child-rated and parent-rated QoL in generic and weight-specific instruments).

**Table 2:** Weight bias and quality of life among participants.

	**Mean (SD)**	**Range (min–max)**	**Possible range**
**WBIS score**	23.22 (8.33)	39 (11–50)	11–55
**WSSQ Q1–6 score**^**a**^	12.51 (4.70)	24 (6–30)	6–30
**WSSQ Q7–12 score**^**b**^	10.94 (4.88)	24 (6–30)	6–30
**EWS score**	1.5 (2.13)	9 (0–9)	0–10
**Child-rated KINDL**			
Physical	71.06 (16.72)	93.75 (6.25–100)	0–100
Emotional	74.74 (17.38)	93.75 (6.25–100)	0–100
Self-esteem	44.83 (22.16)	100 (0–100)	0–100
Family	66.49 (17.86)	100 (0–100)	0–100
Friend	69.90 (18.72)	93.75 (6.25–100)	0–100
School	54.22 (18.74)	100 (0–100)	0–100
**Parent-rated KINDL**			
Physical	73.31 (14.55)	75 (25–100)	0–100
Emotional	72.66 (14.24)	75 (25–100)	0–100
Self-esteem	51.52 (17.48)	100 (0–100)	0–100
Family	70.81 (14.85)	87.5 (12.5–100)	0–100
Friend	68.97 (14.34)	81.25 (18.75–100)	0–100
School	65.48 (14.85)	87.5 (12.5–100)	0–100
**Sizing Me Up**			
Emotional	87.82 (17.98)	100 (0–100)	0–100
Physical	89.80 (15.75)	93.33 (6.67–100)	0–100
Teasing experience	87.93 (18.06)	100 (0–100)	0–100
Positive attributes	38.82 (21.49)	100 (0–100)	0–100
Social avoidance	90.96 (13.71)	73.33 (26.67–100)	0–100
**Sizing Them Up**			
Emotional	91.18 (11.03)	52.38 (47.62–100)	0–100
Physical	94.86 (9.14)	53.33 (46.67–100)	0–100
Teasing experience	94.41 (11.48)	66.67 (33.33–100)	0–100
Positive attributes	50.93 (19.25)	100 (0–100)	0–100
Mealtime disturbance	85.94 (16.48)	83.33 (16.67–100)	0–100
School	98.45 (8.37)	100 (0–100)	0–100

*WBIS, Weight Bias Internalization Scale; WSSQ, Weight Self-Stigma Questionnaire; EWS, Experienced Weight Stigma. KINDL is a generic quality of life instrument; Sizing Me Up and Sizing Them Up are weight-related quality of life instruments*.

a *Indicates self-devaluation domain (i.e., similar to self-stigma)*.

b *Indicates fear of enacted domain (i.e., similar to perceived stigma)*.

**Table 3:** Factor loadings of all instrument items in each proposed model.

**Construct**	**Item**	**Factor loading**			
		**Model 1**	**Model 2**	**Model 3**	**Model 4**
Weight-related self-stigma	WBIS1	0.212	0.200	0.200	0.197
	WBIS2	0.694	0.687	0.689	0.683
	WBIS3	0.739	0.738	0.733	0.735
	WBIS4	0.486	0.489	0.492	0.494
	WBIS5	0.783	0.784	0.782	0.780
	WBIS6	0.767	0.768	0.779	0.771
	WBIS7	0.586	0.600	0.595	0.590
	WBIS8	0.714	0.709	0.711	0.705
	WBIS9	0.503	0.505	0.516	0.518
	WBIS10	0.723	0.721	0.720	0.721
	WBIS11	0.686	0.676	0.675	0.669
	WSSQ1	0.572	0.572	0.557	0.565
	WSSQ2	0.725	0.729	0.722	0.736
	WSSQ3	0.794	0.798	0.797	0.802
	WSSQ4	0.678	0.673	0.667	0.675
	WSSQ5	0.209	0.228	0.219	0.223
	WSSQ6	0.629	0.634	0.636	0.640
Perceived weight stigma	WSSQ7	0.728	0.726	0.711	0.719
	WSSQ8	0.817	0.810	0.820	0.811
	WSSQ9	0.627	0.629	0.628	0.627
	WSSQ10	0.704	0.709	0.711	0.711
	WSSQ11	0.787	0.786	0.788	0.789
	WSSQ12	0.794	0.797	0.801	0.806
Experienced weight stigma	EWS1	0.609	0.625	0.627	0.633
	EWS2	0.642	0.640	0.639	0.634
	EWS3	0.320	0.323	0.337	0.305
	EWS4	0.591	0.578	0.585	0.594
	EWS5	0.667	0.661	0.655	0.660
	EWS6	0.315	0.336	0.346	0.337
	EWS7	0.422	0.423	0.403	0.418
	EWS8	0.587	0.586	0.592	0.590
	EWS9	0.536	0.531	0.520	0.529
	EWS10	0.597	0.587	0.594	0.586
Quality of life	Child KINDL_Phy	0.634	–	–	–
	Child KINDL_Emo	0.694	–	–	–
	Child KINDL_SE	0.375	–	–	–
	Child KINDL_Fam	0.513	–	–	–
	Child KINDL_Fri	0.579	–	–	–
	Child KINDL_Sch	0.603	–	–	–
	Parent KINDL_Phy	–	0.637	–	–
	Parent KINDL_Emo	–	0.719	–	–
	Parent KINDL_SE	–	0.319	–	–
	Parent KINDL_Fam	–	0.622	–	–
	Parent KINDL_Fri	–	0.629	–	–
	Parent KINDL_Sch	–	0.536	–	–
	SMU_Emo	–	–	0.780	–
	SMU_Phy	–	–	0.764	–
	SMU_Tease	–	–	0.673	–
	SMU_Pos	–	–	0.142	–
	SMU_Soc	–	–	0.768	–
	STU_Emo	–	–	–	0.772
	STU_Phy	–	–	–	0.664
	STU_Tease	–	–	–	0.673
	STU_Pos	–	–	–	0.280
	STU_Meal	–	–	–	0.425
	STU_Sch	–	–	–	0.231

*WBIS, Weight Bias Internalization Scale; WSSQ, Weight Self-Stigma Questionnaire; EWS, Experienced Weight Stigma; KINDL, generic quality of life questionnaire; SMU, Sizing Me Up; STU, Sizing Them Up; Phy, physical; Emo, emotional; SE, self-esteem; Fam, family; Fri, friend; Sch, school; Tease, teasing experience; Pos, positive attributes; Soc, social avoidance; Meal, mealtime disturbance*.

*Model 1 applies child-rated KINDL in the model; Model 2 applies parent-rated KINDL in the model; Model 3 applies child-rated Sizing Me Up in the model; Model 4 applies parent-rated Sizing Them Up in the model*.

**Table 4:** Pearson correlation matrix in the studied variables.

	**1**.	**2**.	**3**.	**4**.	**5**.	**6**.	**7**.	**8**.	**9**.
**1. Age**	–								
**2. Gender**	−0.04	–							
**3. BMI**	0.13^**^	−0.01	–						
**4. EWS**	−0.06	−0.05	0.05	–					
**5. PWS**	−0.12^*^	0.02	0.16^**^	0.40^***^	–				
**6. WSS**	−0.10^*^	0.06	0.25^***^	0.39^***^	0.81^***^	–			
**7. Child-rated Kid-KINDL**	−0.07	−0.01	−0.05	−0.47^***^	−0.33^***^	−0.38^***^	–		
**8. Parent-rated Kid-KINDL**	−0.09	−0.004	−0.13^**^	−0.17^***^	−0.19^***^	−0.21^***^	0.37^***^	–	
**9. Sizing Me Up**	0.01	0.01	−0.25^***^	0.43^***^	−0.56^***^	−0.60^***^	0.49^***^	0.33^***^	–
**10. Sizing Them Up**	0.08	−0.02	−0.26^***^	−0.21^***^	−0.38^***^	−0.34^***^	0.13^**^	0.48^***^	0.45^***^

*EWS, experienced weight stigma; PWE, perceived weight stigma; WSS, weight-related self-stigma*.

* p < 0.05;

** p < 0.01;

*** *p < 0.001*.

All the SEM models had satisfactory fit indices (Figure 1). Additionally, experienced weight stigma was significantly associated with perceived weight stigma, and in turn, perceived weight stigma was significantly associated with weight-related self-stigma. However, experienced weight stigma did not directly associate with weight-related self-stigma.

The mediation models (Table 5) showed that experienced weight stigma was not a significant mediator in the association between body weight and children's QoL whether child-rated, parent-rated, weight-related, or generic. The other two types of weight stigma (perceived weight stigma and weight-related self-stigma) were significant mediators in the association between body weight and children's QoL whether child-rated, parent-rated, weight-related, or generic.

**Table 5:** Mediation models testing the indirect effect of different types of weight stigma in the association between body weight and quality of life.

**Mediator**	**Coefficient (Bootstrapping SE)**	**Bootstrapping LLCI**	**Bootstrapping ULCI**
**Dependent variable: Child-rated Kid-KINDL**			
EWS	−0.08 (0.06)	−0.21	0.04
PWS	−0.18 (0.06)	−0.33	−0.08
WSS	−0.32 (0.09)	−0.51	−0.17
**Dependent variable: Parent-proxy Kid-KINDL**			
EWS	−0.25 (0.11)	−0.07	0.13
PWS	−0.08 (0.04)	−0.16	−0.03
WSS	−0.14 (0.04)	−0.24	−0.06
**Dependent variable: Sizing Me Up**			
EWS	−0.07 (0.06)	−0.18	0.03
PWS	−0.26 (0.10)	−0.48	−0.10
WSS	−0.42 (0.11)	−0.65	−0.24
**Dependent variable: Sizing Them Up**			
EWS	−0.02 (0.02)	−0.07	0.01
PWS	−0.12 (0.05)	−0.23	−0.04
WSS	−0.15 (0.04)	−0.25	−0.08

*Age and gender were controlled in all the mediation models. Hayes' Process macro (Model 4) was used; each mediation model generated 5,000 bootstrapping samples*.

*EWS, experienced weight stigma; PWE, perceived weight stigma; WSS, weight-related self-stigma; LLCI, 95% lower limit of confidence interval; ULCI, 95% upper limit of confidence interval*.

## Discussion

Although weight-related stigma and QoL research are prevalent in the literature, no studies have examined the different types of weight-related stigma and their combined effects on different types of QoL. The current study used psychometrically sound assessments on 430 dyads of 8- to 12-year-old children and their parents to construct four models investigating different types of weight-related stigma on children's QoL. Congruent with the first hypothesis, children's experienced stigma was positively associated with their perceived stigma, and then the perceived stigma was further positively associated with children's self-stigma. Aligning with the second hypothesis, the experienced stigma was the primary stigma that negatively associated with both child-rated and parent-rated generic QoL and weight-related QoL. Perceived weight stigma was associated with only parent-rated weight-related QoL but not any of child-rated QoL. Self-stigma associated with both child-rated generic QoL and weight-based QoL but not any of parent-rated QoL. Consistent with the third hypothesis, perceived weight stigma and weight-related self-stigma were significant mediators in the association between body weight and QoL; however, experienced weight stigma was not. Further explanations are illustrated below.

### Experienced Weight Stigma, Perceived Weight Stigma, and Weight-Related Self-Stigma

Congruent with Gmeiner's and Warschburger's (2020) previous evidence, the present study found that children's experienced weight stigma was positively associated with their perceived weight stigma, and perceived stigma was, in turn, associated with their weight-related self-stigma. Gmeiner and Warschburger (2020) conducted a longitudinal study and found that when children experienced more weight-related teasing, they have increased weight-related self-stigma. This association also agrees with the previous framework on how self-stigma is formed in people with mental illness. People with mental illness first experience unfriendly treatment (i.e., experienced stigma), then become aware that the stigma is due to their characteristics (i.e., perceived stigma). After recognizing the stigma, they may accept and endorse the biased attitudes and treatments they experienced and develop self-stigma (Lin et al., 2016). Thus, our findings echo and support this framework that children with weight concerns may develop weight-related self-stigma *via* the same pathways; that is, weight-related self-stigma is developed from experienced stigma and perceived stigma.

### Experienced Weight Stigma and Quality of Life

Among the three types of weight-related stigma, experienced stigma played a critical role and was negatively associated with both child-rated and parent-rated QoL (in both generic and weight-related QoL). Such a result is consistent with previous findings that experienced stigma is associated with lower psychological QoL (Greenleaf et al., 2014) and poor psychological functioning (Gunnarsdottir et al., 2012; Zuba and Warschburger, 2017), and it especially severely affected the youth population (Pont et al., 2017). Multiple studies found that individuals who experience weight stigma have poorer long-term weight loss outcomes than those who do not experience stigma (Carels et al., 2009; Wott and Carels, 2010; Gudzune et al., 2014). Additionally, experienced stigma contributes to problematic consequences such as unhealthy eating behaviors, social isolation, avoidance of health care services, decreased exercise, and impaired QoL (Pont et al., 2017).

Furthermore, a study reviewed 15 prospective cohort studies and found that children with obesity were about five times more likely to be with obesity in adulthood than those who were not with obesity (Simmonds et al., 2016). Therefore, once manifested, children who experience weight-related stigma may remain such uncomfortable feelings until adulthood, hence increasing the risk of developing chronically psychological distress and lowered QoL (Parker and Aggleton, 2003; Palad et al., 2019).

However, experienced weight stigma was found to be a non-significant mediator in the association between body weight and QoL, which is inconsistent with findings by Guardabassi et al. (2018). Closely scrutinizing the assessments used in their study, they adopted the Perception of Teasing Scale, which had 11 items rated on a 5-point Likert scale, to evaluate children's experienced stigma (Guardabassi et al., 2018). Our study used the EWS questionnaire, which collected children's experienced stigma with a dichotomous classification (Yes/No). Therefore, EWS potentially did not capture all the different experienced weight stigma levels, which might reduce the power of experienced stigma to serve as a significant mediator. Nevertheless, our non-significant result is consistent with that of a previous study on an American adult population, in which experienced stigma was not a significant mediator of the relationship between BMI and psychical and psychological health (Hunger and Major, 2015). Future studies may need to examine further whether our postulation that the use of different instruments on experienced weight stigma really matters in the mediational relationship.

### Perceived Weight Stigma and Quality of Life

In the current study, we found that perceived weight stigma was associated with neither child-rated generic QoL nor weight-related QoL, but only parent-rated weight-related QoL. Perceived weight stigma is highly influenced by social environments (Puhl and Heuer, 2010). For example, children may perceive that their own body image aligns with social media exposure and parental behaviors and perceptions (Robinson et al., 2017). The investigation by Puhl et al. (2016) on parental perceptions of weight-based stigma showed that “being overweight” was perceived as the most common and substantial concern that parents perceived for their children being stigmatized regardless of their weight status. Interestingly, a multinational study indicated that about 70% of participants (*n* = 2,866) across four countries (i.e., United States, Canada, Iceland, and Australia) perceived weight-related stigma as the most common and serious problem in child populations (Puhl et al., 2016). Robinson et al. (2017) found that parents' perception of their children as overweight increases the likelihood that children will negatively view and recognize their body size. Additionally, compared with parents without concern for their children's weight, parents who had concerns were identified with significantly lower perceived self-efficacy (Klupt et al., 2020), which may unavoidably influence the children's own weight-related perceptions. Therefore, it is essential to understand the parent's influence on profound perceptions when considering why the perceived stigma is positively correlated with parent-rated weight-related QoL.

In contrast, a previous study indicated that children might have different sensitivity in perceived stigma; some might be more vulnerable to weight-related oppression than others (Puhl and Heuer, 2009). A review (Mak et al., 2007) further showed that the stigmatized individuals' QoL and mental health would only be affected if they perceive the negative stereotypes or discrimination toward them as legitimate. More recent studies discussed weight bias as an important social justice issue to be addressed in research, policy, and practice (Cardinal et al., 2014; Nutter et al., 2016). Therefore, given that the social atmosphere has been established to consider the influence of weight bias on social inequity, stigmatized children are more likely to show righteous anger toward perceived stigma when the negative acts are viewed as not legitimate, resulting in a non-significant difference in their QoL (Mak et al., 2007).

The mediation model in our analysis further showed that perceived stigma is a significant mediator between body weight and QoL. This finding aligns with that of a previous study that the relationship between higher BMI and poorer psychological health is indirect, mediated by increased perceived weight stigma in an American adult population (Hunger and Major, 2015). This pattern is also consistent with other recent evidence using data from three large samples of predominantly US and UK adults that weight status and depressive symptoms were in part explained by the subjects' perceived weight stigma (Robinson et al., 2017).

### Weight-Related Self-Stigma and Quality of Life

Our results showed that weight-related self-stigma was associated with both child-rated generic QoL and weight-based QoL but not any parent-rated QoL. Consistent with previous studies' findings showing the relationship between self-stigma and QoL among an adult population (Latner et al., 2013; Kahan and Puhl, 2017), the present study demonstrated the significant association between self-stigma and QoL, thus filling the literature gap (Pearl and Puhl, 2018). In a systematic review that examined associations between self-stigma and different QoL domains, the results showed significant negative relationships between self-stigma and psychological domains of QoL (Pearl and Puhl, 2018). Another study examined the potential moderating role of self-stigma in the association between BMI and QoL (Latner et al., 2014) in 81 women. The results indicated a strong association between BMI and QoL's physical domains only exists for individuals with high self-stigma but not for individuals with low self-stigma. Zuba and Warschburger (2017) conducted a longitudinal study to examine self-stigma in children aged 7–11 years old with various weight statuses. The results suggested that self-stigma mediated the relationship between BMI and emotional problems, and self-stigma is more important than weight status in explaining psychological functioning. The present study's results are consistent with those of Wong et al. (2019) that children with obesity had significantly higher self-stigma and lower QoL than children without obesity. Additionally, underlying parental attitudes may increase the risk of children's lowered esteem and results in children's self-stigma and lowered QoL (Lydecker et al., 2018). Therefore, it highlighted the unique role of self-stigma on QoL.

Additionally, we found that weight-related self-stigma was a significant mediator in the association between body weight and children's QoL. Several studies have conducted mediational analyses to investigate the association between self-stigma and QOL in an adult population. For example, Palmeira et al. (2019) examined the effectiveness of a group intervention on 53 women with overweight and obesity. They found that self-stigma was an important mediator of QoL changes. Another study on 1,158 German adults found that self-stigma significantly predicted lower QoL, and they identified self-compassion as a major psychological resource that mediated the self-stigma process (Hilbert et al., 2015). Another study recruited 87 adults from a weight loss clinic and found that self-stigma was a significant predictor of HRQoL and mediated the relationship between BMI and HRQoL (Lillis et al., 2011). Also, Pearl et al. (2014) found that self-stigma mediated the relationship between depression and QoL.

The discrepancy between child-rated and parent-rated QoL was found in the literature concerning the perceived stigma and self-stigma being inconsistently associated with child-rated and parent-rated QoL. A review article examined 19 studies that included four QoL instruments. The results confirmed that there were differences in parent–child agreement across domains for different QoL measurements (Upton et al., 2008). Another study further found that parents of healthy children tended to report higher QoL than their children did. In contrast, parents of children with health conditions tended to underestimate their children's QoL (Tsiros et al., 2009), particularly among parents of young children with obesity (Ruiter et al., 2020). Also, cross-sectional community studies from Spain (Herranz Barbero et al., 2013), Australia (Wake et al., 2002), Taiwan (Lin et al., 2013; Su et al., 2013), and Hong Kong (Fung, 2018) have examined the discrepancy between child-rated and parent-rated QoL in children. The results supported that parents seemed to be optimistic when rating the QoL of their children with obesity and tended to overestimate their QoL. However, another study conducted by Jafari et al. (2016) found the opposite—that Iranian parents of children with obesity rated the child's QoL significantly lower than their children did. Therefore, considering the inconsistency between child-rated and parent-rated QoL is critical. It is suggested that clinicians do not use parent-proxy assessment alone to evaluate the QoL for children with obesity or overweight because parents may overestimate or underestimate their children's difficulties.

### Limitations, Strengths, and Future Directions

The study has several limitations. First, the children and their parents in the study voluntarily participated, which may have biased our results. Second, all questionnaires used in the current study were self-reported. Although we attempted to maximize participants' honest responses, inevitably, social desirability and recall bias might have influenced the study results. Third, the assessment used to collect the children's experienced stigma used a dichotomous classification; therefore, it may be unable to well-capture the different levels of experienced weight stigma. This possible deficiency should be considered when interpreting the findings. Fourth, the study design's correlational nature cannot draw conclusions about the causal relationship among the different types of weight-related stigma and QoL. Last, the study used a convenience sampling and only enrolled participants from Hong Kong, which limited its generalizability to other geographic locations.

Despite these limitations, the study has several strengths. First, to the best of our knowledge, this study is the first to simultaneously examine the associations between different types of weight stigma and different types of QoL. The present findings concur with prior evidence to demonstrate the associations between weight stigma and QoL. The three types of weight stigma correlated with each other and made specific contributions to the QoL when the effects of other types of weight stigma were taken into consideration. Second, the weight stigma's mediated effects were clearly demonstrated and supported by the present study's results. Third, the present study focused on a unique population, i.e., children, to supplement the weight stigma research in this specific population.

The limitations and strengths of the present study suggest several avenues for more in-depth studies in the future. First, given the present study's cross-sectional design, the proposed mediational models showed little evidence of their causal relationships. Therefore, adopting a longitudinal design in future studies is warranted to corroborate the present study's proposed mediational models. Second, the association between weight stigma and QoL is supported, but it is unclear whether reducing weight stigma can improve QoL in a pediatric population. Therefore, future studies are needed to test such treatment effects.

## Conclusion

In conclusion, the study findings support that the experienced stigma is associated with perceived stigma, and the perceived stigma is associated with self-stigma. Additionally, all types of weight-related stigma are associated with children's QoL, while different types of stigma have varied levels of effects on child-rated and parent-rated QoL. Most importantly, the present study found that perceived weight stigma and weight-related self-stigma (but not experienced weight stigma) significantly mediate the relation between body weight and children's QoL.

## Data Availability Statement

The raw data supporting the conclusions of this article will be made available by the authors, without undue reservation.

## Ethics Statement

The studies involving human participants were reviewed and approved by The Human Subject Ethics Review Board in the Hong Kong Polytechnic University (Ref No: HSEARS20160824003) approved the study proposal before data collection commenced. Written informed consent to participate in this study was provided by the participants' legal guardian/next of kin.

## Author Contributions

C-WF, C-hL, and C-YL created and organized the study. C-YL collected the data. H-HH analyzed the data. C-WF, AP, C-hL, and C-YL wrote the first draft and analyzed and interpreted the data. AP, C-YL, C-hL, H-HH, and C-WF critically reviewed the manuscript and provided constructive comments. All authors contributed to the article and approved the submitted version.

## Conflict of Interest

The authors declare that the research was conducted in the absence of any commercial or financial relationships that could be construed as a potential conflict of interest.

## Abbreviations

BMI: Body mass index
QoL: Quality of life
HRQoL: Health-related quality of life
NGO: Non-government organization
WBIS: Weight Bias Internalization Scale
WSSQ: Weight Self-Stigma Questionnaire
EWS: Experienced weight stigma
SMU: Sizing Me Up
STU: Sizing Them Up
HKD: Hong Kong Dollar
SEM: Structural equation modeling
DWLS: Diagonally weighted least squares
CFI: Comparative fit index
TLI: Tucker-Lewis index
RMSEA: Root mean square error of approximation
SRMR: Standardized root mean squared residual.
